# Why does LDL-C follow a different paradigm from other endocrine–metabolic diseases in cardiovascular prevention?

**DOI:** 10.3389/fendo.2026.1813342

**Published:** 2026-04-27

**Authors:** José Luis Sánchez-Quesada, Antonio Pérez

**Affiliations:** 1 Cardiovascular Biochemistry, Institut de Recerca Sant Pau (IR Sant Pau), Barcelona, Spain; 2 CIBER of Diabetes and Associated Metabolic Diseases (CIBERDEM), Barcelona, Spain; 3 Department of Endocrinology and Nutrition, Hospital de la Santa Creu i Sant Pau, Barcelona, Spain; 4 Facultat de Medicina, Universitat Autònoma de Barcelona, Barcelona, Spain

**Keywords:** cardiovascular prevention, cumulative exposure, dyslipidaemia, LDL cholesterol, lifetime risk

## Abstract

Cardiovascular prevention has traditionally focused on achieving progressively lower low-density lipoprotein cholesterol (LDL-C) targets, particularly in individuals classified as high risk. However, accumulating evidence from epidemiological, genetic and clinical studies suggests that cardiovascular risk is strongly influenced by cumulative lifetime exposure to LDL-C rather than by isolated LDL-C levels achieved later in life. From an endocrine perspective, LDL-C should be viewed as a chronic metabolic exposure whose pathogenic impact depends not only on its intensity, but also on its duration over time. This Perspective integrates evidence from longitudinal cohort studies, familial hypercholesterolaemia and Mendelian randomisation analyses, and contrasts current lipid management paradigms with preventive strategies routinely applied to other endocrine–metabolic disorders. While contemporary guidelines recognise the causal role of LDL-C and increasingly acknowledge lifetime risk, this recognition is not always fully translated into operational criteria for early intervention, particularly in younger adults. Within this context, earlier approaches aimed at limiting cumulative LDL-C exposure may offer additional long-term benefit, especially in individuals with long life expectancy. This Perspective, grounded in an endocrine framework, is intended to complement, rather than replace, current guideline-based strategies by highlighting the potential value of incorporating cumulative exposure into preventive decision-making.

## 1 An endocrine inconsistency in cardiovascular prevention

From a classical endocrine perspective, effective prevention is based on the early and sustained correction of causal metabolic exposures before irreversible organ damage develops. This principle is consistently applied to hyperglycaemia, hormonal excess or deficiency, and pathological adiposity, where early intervention aims to minimise cumulative exposure and prevent structural complications. It is therefore striking that this same reasoning is not applied with comparable coherence to chronic exposure to low-density lipoprotein cholesterol (LDL-C), despite the fact that its causal relationship with atherosclerotic cardiovascular disease (ASCVD) is firmly established through genetic, epidemiological and interventional evidence (1, 2).

From a pathophysiological standpoint, atherosclerosis represents the biological consequence of prolonged exposure to circulating atherogenic lipoproteins. Cholesterol accumulation within the arterial wall originates predominantly from LDL particles and progressively drives plaque formation over decades. In this context, LDL-C should be regarded not merely as a risk marker, but as a direct substrate for disease development, making duration of exposure a central determinant of atherogenesis.

Despite this biological framework, contemporary cardiovascular prevention has increasingly emphasized short-term risk estimation and the detection of established vascular disease over etiological correction. This approach may have important implications, particularly in younger adults, in whom short-term cardiovascular risk is intrinsically low, while cumulative exposure to LDL-C may already be substantial. As a result, preventive strategies tend to be more reactive, with intervention often deferred until risk becomes numerically elevated or disease becomes detectable, rather than addressing the atherogenic process during its earliest and most modifiable phases.

## 2 Parallels with endocrine preventive strategies

When viewed from an endocrine perspective, this approach to LDL-C management contrasts sharply with preventive strategies routinely applied in other endocrine–metabolic disorders. In diabetes, sustained hyperglycaemia is treated long before retinopathy or nephropathy develops (3, 4); endogenous hypercortisolism is corrected without waiting for fractures or overt cardiovascular disease (5); and hyperthyroidism is treated before atrial fibrillation or heart failure emerges (6). In these contexts, early intervention is not discretionary, but a fundamental principle of prevention.

A unifying concept across endocrinology is that cumulative exposure—rather than isolated biochemical values—determines long-term organ damage. The duration of metabolic or hormonal imbalance is often more predictive of outcomes than severity at diagnosis, and preventive strategies are explicitly designed to limit exposure over time.

By contrast, when the chronic exposure in question is LDL-C, intervention is frequently postponed until vascular injury becomes evident. In younger adults, the absence of detectable atherosclerosis is therefore often interpreted as biological safety rather than recognised as a period of high preventive potential. This divergence highlights a difference in how preventive reasoning is operationalised across clinical domains and provides the conceptual framework for examining how current cardiovascular guidelines implement risk and treatment decisions.

## 3 Guideline-driven prevention and the primacy of short-term risk

This conceptual divergence is not merely theoretical, but is explicitly embedded in the operational structure of contemporary cardiovascular prevention guidelines. Despite formal recognition of LDL-C as a causal factor in ASCVD and repeated acknowledgment of lifetime risk, most major guidelines continue to organise treatment decisions predominantly around short-term risk estimation and evidence of established vascular injury.

The ESC/EAS dyslipidaemia guidelines prioritise 10-year cardiovascular risk assessment using SCORE-based models and promote risk reclassification based on markers of manifest atherosclerosis, such as carotid or femoral plaque or coronary artery calcium (7). Similarly, the ACC/AHA cholesterol and primary prevention guidelines frame statin initiation around pooled cohort equations and endorse coronary artery calcium scoring as a “reasonable” tool to resolve therapeutic uncertainty (8, 9). The 2022 ACC Expert Consensus Decision Pathway further reinforces this approach by considering earlier LDL-C intervention in younger adults mainly in the presence of risk enhancers, genetic dyslipidaemia or imaging findings suggestive of subclinical disease (10).

Other influential organisations adopt comparable frameworks. NICE recommendations continue to rely primarily on estimated 10-year cardiovascular risk thresholds to guide lipid-lowering therapy in primary prevention (11), while AACE and Endocrine Society guidelines more explicitly recognise the etiological role of dyslipidaemia and the relevance of lifetime risk, yet still depend largely on short-term risk stratification in clinical implementation (12, 13).

As a result, although the causal and cumulative nature of LDL-C exposure is conceptually acknowledged across guidelines, this knowledge is not always systematically translated into operational criteria for early intervention, particularly in adults under 40 years of age. In clinical practice, treatment initiation in this population is frequently deferred until short-term risk thresholds are exceeded or structural vascular disease becomes evident, thereby allowing prolonged exposure to atherogenic LDL-C to persist before intervention is considered. Taken together, contemporary guidelines clearly recognise LDL-C as a causal factor and increasingly acknowledge the importance of lifetime exposure. However, this conceptual recognition is not always fully translated into operational criteria for early intervention, particularly in adults under 40 years of age. In clinical practice, treatment decisions in this population are often guided by short-term risk thresholds or evidence of established disease. A comparative overview of how major guideline frameworks operationalise LDL-C management in adults under 40 years is summarised in **Table 1**.

**Table 1:** Guideline perspectives on LDL-C management in adults <40 years.

Guideline (year)	Core recommendation in adults <40 years	Operational focus	Authors’ perspective
ESC/EAS Dyslipidaemia Guidelines (2019; subsequent conceptual updates)	Recognise LDL-C as a causal factor and acknowledge lifetime exposure; treatment mainly guided by 10-year SCORE risk and presence of subclinical atherosclerosis	Short-term risk estimation; imaging-based risk reclassification	Strong conceptual recognition of cumulative exposure, but inconsistently operationalised in younger adults; early intervention remains largely discretionary
ACC/AHA Cholesterol Guideline (2018)	Statin therapy recommended for LDL-C ≥190 mg/dL or based on pooled cohort equations; limited specific guidance for adults <40 years	Threshold-based, short-term risk driven	Establishes the causal role of LDL-C but operationalises treatment mainly through threshold-based criteria, with many young adults with moderate elevations not routinely considered for treatment
ACC/AHA Primary Prevention Guideline (2019)	Emphasises risk enhancers and shared decision-making; coronary artery calcium scoring endorsed to refine decisions	Risk estimation and imaging to resolve uncertainty	Reinforces a predominantly reactive prevention model, with intervention often deferred until risk becomes quantifiable or disease detectable
ACC Expert Consensus Decision Pathway (2022)	Allows earlier intervention in selected younger adults with risk enhancers or genetic dyslipidaemia	Risk enhancers and imaging-based triggers	Introduces greater flexibility, but still does not systematically translate cumulative exposure into treatment decisions,
NICE Cardiovascular Risk and Lipid Modification (2023)	Statin therapy guided by estimated 10-year risk; clinician discretion permitted in younger adults	Pragmatic short-term risk thresholds	Offers some flexibility for earlier intervention, but does not explicitly incorporate age- or cumulative exposure-based operational criteria
AACE Dyslipidaemia Guidelines (2020)	Recognises lifetime ASCVD risk and aetiological dyslipidaemia; supports earlier consideration of treatment	Risk stratification with emphasis on global risk	Most explicitly aligned with an exposure-based paradigm, though implementation still largely relies on short-term risk
Endocrine Society Lipid Management Guideline (2020)	Frames dyslipidaemia as a causal metabolic disorder; supports proactive management	Aetiological and metabolic focus	Closest alignment with endocrine preventive principles, but without clearly defined operational thresholds for adults <40 years

The “Authors’ perspective” column reflects the authors’ contextual interpretation of how current guideline recommendations relate to the conceptual framework discussed in this article and is intended as an interpretative and contextual synthesis rather than a direct or exhaustive representation of individual guideline positions.

This disconnect between conceptual recognition and clinical implementation provides the critical context for re-examining current prevention strategies. If LDL-C is accepted as a causal, cumulative exposure across the life course, then deferring intervention until short-term risk becomes elevated or vascular damage is detectable may reflect an inherent limitation of current prevention frameworks, which must balance causal reasoning with available trial evidence, treatment burden, long-term safety, adherence, and the relevance of short-term absolute risk.

## 4 A time-dependent model of LDL-C–related risk

Precisely because current guidelines and clinical practice continue to prioritise short-term risk estimation and evidence of vascular injury, it is important to emphasise that the scientific evidence consistently supports the concept of a model of cardiovascular risk that is fundamentally dependent on time and cumulative exposure to LDL-C.

Longitudinal cohort studies have demonstrated that exposure to elevated LDL-C from early adulthood is independently associated with a progressive increase in future ASCVD risk, even after adjustment for LDL-C levels measured later in life. This indicates that cardiovascular risk is determined by the history of exposure rather than by isolated lipid measurements (14, 15). Importantly, analyses of LDL-C trajectories further show that accumulating a given LDL-C burden earlier in life confers greater cardiovascular risk than achieving the same cumulative exposure later, underscoring that the timing of exposure reduction is a critical determinant of long-term benefit (16).

This time-dependent model is reinforced by converging genetic and clinical evidence. Mendelian randomisation studies demonstrate that lifelong or early genetically mediated reductions in LDL-C are associated with approximately 50–55% reductions in coronary risk per 1 mmol/L decrease, an effect size substantially greater than that observed when comparable LDL-C lowering is initiated pharmacologically in adulthood (17, 18).

Familial hypercholesterolaemia (FH) provides direct clinical validation of this concept. Intervention studies in children and adolescents with heterozygous FH have shown that early initiation of statin therapy slows the progression of subclinical atherosclerosis and is associated on long-term follow-up with a marked reduction in cardiovascular events in adulthood, approaching the risk observed in the general population when treatment begins early (19, 20). Notably, these benefits are achieved without the need for extreme LDL-C targets, but through sustained moderate reductions initiated at young ages, underscoring that early control of exposure is more decisive than late intensive lipid lowering.

Taken together, epidemiological, genetic and clinical data converge on a unified model in which cardiovascular risk is determined by the cumulative burden of LDL-C exposure over time, and in which earlier intervention yields disproportionately greater lifetime benefit than aggressive treatment initiated after decades of atherogenic exposure.

## 5 Deficits of current prevention and efficiency of early LDL-C intervention

**Figure 1:**
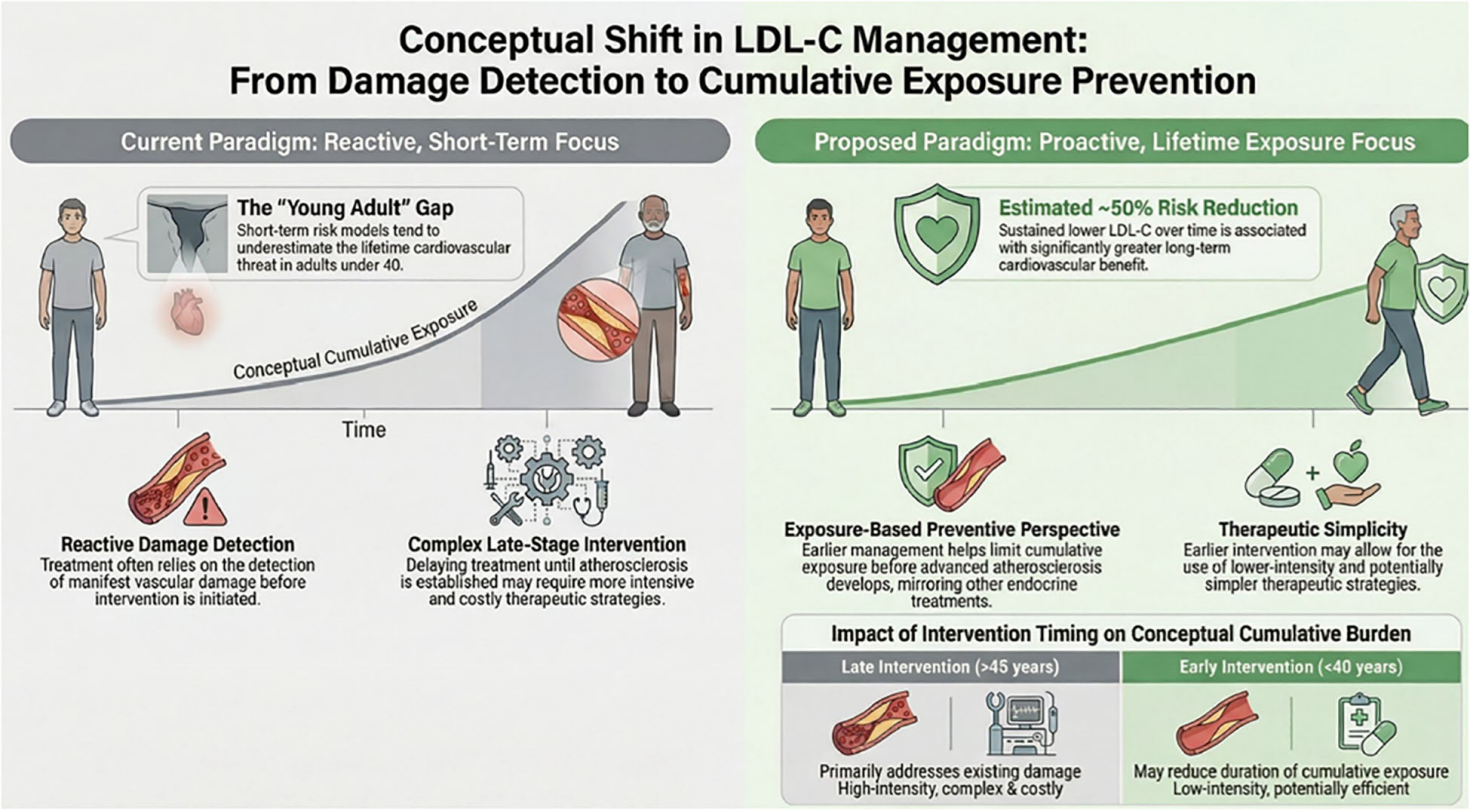
This figure is intended as a conceptual and illustrative model of cumulative LDL-C exposure over time. It should not be interpreted as a quantitative estimate of risk or as a direct representation of clinical decision thresholds. The elements presented aim to support interpretation of cumulative exposure rather than to define specific treatment strategies.

Within this framework, the central limitation of contemporary cardiovascular prevention lies not in insufficient evidence or therapeutic capability, but in the persistent delay in addressing LDL-C as a causal exposure over time. Accepting prolonged exposure to moderately elevated LDL-C in the context of low short-term risk may reflect the need to balance current evidence, feasibility, long-term safety considerations and treatment burden, while also indicating that cumulative exposure is not yet fully integrated into preventive decision-making. This conceptual shift can be illustrated through a simplified representation of cumulative LDL-C exposure over time (**Figure 1**).

Several authors have therefore proposed reconsidering both age thresholds and LDL-C cut-offs for treatment initiation, including in young adults with so-called borderline LDL-C levels, based on cumulative exposure, greater lifetime absolute benefit and favourable cost-effectiveness (21, 22). This approach should not be interpreted as aggressive or disproportionate, but as an attempt to normalise a chronic pathological exposure before irreversible vascular damage develops.

For young individuals (typically <40–45 years) with elevated LDL-C and long life expectancy, the preventive objective should not be confined to reducing 10-year cardiovascular risk, but rather to minimising total LDL-C exposure over time. The earlier LDL-C lowering is initiated, the more important it becomes to maintain lower levels for longer durations. Accordingly, the same absolute LDL-C reduction sustained over 30–40 years (for example, from age 20 to 60) yields a far greater reduction in cardiovascular risk than an identical reduction initiated only after age 40–45. From a cholesterol burden perspective, intervening before age 20 nearly halves the cumulative years of exposure to elevated LDL-C compared with intervention initiated after age 40 (14–16).

In clinical practice, this conceptual framework may translate into earlier clinical recognition of dyslipidaemia as an etiological diagnosis, particularly in younger adults with persistent LDL-C elevations (typically >130 mg/dL and especially in the 160–190 mg/dL range), where the probability of an underlying lipid disorder is higher. In such individuals, consideration of lifetime LDL-C exposure may support earlier clinical attention and, where appropriate, the use of moderate, well-tolerated lipid-lowering strategies aimed at limiting cumulative exposure rather than waiting for short-term cardiovascular risk thresholds to be exceeded. Despite this evidence, in clinical practice young adults with persistently elevated LDL-C are commonly managed with observation, generic lifestyle advice or postponement of therapeutic decisions until short-term cardiovascular risk reaches a “sufficiently high” threshold. This strategy, deeply rooted in short-term risk stratification, tends to underestimate lifetime risk and may result in preventive interventions being initiated at stages when arterial disease is already present (1, 2).

From both a clinical and health-economic perspective, timing has direct implications for preventive efficiency. Early LDL-C intervention allows meaningful lifetime risk reduction to be achieved with low- or moderate-intensity statin therapy, which is effective, well tolerated and inexpensive. By reducing cumulative atherogenic burden early in life, this strategy limits the biological substrate upon which atherosclerosis develops and aligns biological efficiency with therapeutic simplicity. In contrast, delaying intervention until atherosclerosis is established often necessitates increasingly intensive lipid-lowering strategies, including combination therapy and novel agents, to achieve very low LDL-C targets. These approaches are more complex and costlier, while delivering smaller absolute benefits constrained by prior irreversible exposure (21, 22).

Preventive strategies extending over several decades inevitably raise practical considerations related to treatment adherence, patient acceptance and perceived treatment burden. However, statins have demonstrated a favourable long-term safety profile in both randomised clinical trials and extensive real-world experience. When lipid-lowering therapy is considered in individuals with persistent LDL-C elevation and high lifetime cardiovascular risk, the available evidence suggests that the long-term balance between benefit and potential treatment burden is likely to remain favourable, particularly when treatment decisions are individualised and discussed with patients.

Taken together, these considerations indicate that the limitations of current LDL-C prevention strategies are not merely quantitative, but fundamentally conceptual, setting the stage for a necessary shift from damage detection to exposure prevention.

## 6 Conclusions: from detecting damage to preventing exposure

From an endocrine perspective, persisting with a preventive model that requires either high short-term risk or demonstrable vascular damage before intervening cannot be interpreted as clinical prudence, but rather as preventive resignation. In other endocrine–metabolic conditions, sustained pathological exposures are treated early and proactively to prevent irreversible complications; the rationale for applying a different standard to chronic LDL-C exposure deserves closer consideration.

This shift in perspective is particularly relevant in young adults with type 1 diabetes and other populations characterised by a high lifetime cardiovascular risk, who frequently present persistent moderate LDL-C elevation, a strong family history of premature ASCVD, early metabolic dysfunction or genetically mediated lifelong LDL-C exposure, yet remain classified as low risk by short-term algorithms (23, 24). In such settings, deferring intervention until imaging evidence of disease emerges or short-term cardiovascular risk becomes overtly elevated may reduce the opportunity to intervene during the period of greatest preventive potential.

This perspective is intended to complement, rather than replace, current guideline-based strategies. From this standpoint, prevention may be more effective when focused not only on early detection, but also on limiting cumulative exposure at its biological origin.

## Data Availability

The original contributions presented in the study are included in the article/supplementary material. Further inquiries can be directed to the corresponding author.
